# Investigating Master–Slave Architecture for Underwater Wireless Sensor Network

**DOI:** 10.3390/s21093000

**Published:** 2021-04-25

**Authors:** Sadeeq Jan, Eiad Yafi, Abdul Hafeez, Hamza Waheed Khatana, Sajid Hussain, Rohail Akhtar, Zahid Wadud

**Affiliations:** 1 Department of Computer Science and IT, University of Engineering and Technology Peshawar, Peshawar 25000, Pakistan; sadeeqjan@uetpeshawar.edu.pk; 2 Malaysian Institute of Information Technology, Universiti Kuala Lumpur, Kuala Lumpur 50250, Malaysia; 3 Department of Computer Systems Engineering, University of Engineering and Technology Peshawar, Peshawar 25000, Pakistan; abdul.hafeez@uetpeshawar.edu.pk (A.H.); 15pwcse1348@uetpeshawar.edu.pk (H.W.K.); 15pwcse1354@uetpeshawar.edu.pk (S.H.); 15pwcse1360@uetpeshawar.edu.pk (R.A.); zahidmufti@nwfpuet.edu.pk (Z.W.)

**Keywords:** underwater wireless sensor networks, energy optimization, packet duplication, master–slave architecture, depth-based routing

## Abstract

A significant increase has been observed in the use of Underwater Wireless Sensor Networks (UWSNs) over the last few decades. However, there exist several associated challenges with UWSNs, mainly due to the nodes’ mobility, increased propagation delay, limited bandwidth, packet duplication, void holes, and Doppler/multi-path effects. To address these challenges, we propose a protocol named “An Efficient Routing Protocol based on Master–Slave Architecture for Underwater Wireless Sensor Network (ERPMSA-UWSN)” that significantly contributes to optimizing energy consumption and data packet’s long-term survival. We adopt an innovative approach based on the master–slave architecture, which results in limiting the forwarders of the data packet by restricting the transmission through master nodes only. In this protocol, we suppress nodes from data packet reception except the master nodes. We perform extensive simulation and demonstrate that our proposed protocol is delay-tolerant and energy-efficient. We achieve an improvement of 13% on energy tax and 4.8% on Packet Delivery Ratio (PDR), over the state-of-the-art protocol.

## 1. Introduction

Underwater Sensor Networks (UWSNs) have emerged as a field of research in recent years due to their applications for various purposes, e.g., monitoring water pollution, water quality testing, aquaculture, and exploration of geological resources (gas, oil, etc.). Such networks are composed of battery-powered nodes stationed in water to sense the environmental parameters, send them onwards to sink nodes floating on water and pass data to the monitoring station. These nodes can communicate using electromagnetic/radio signals, optical communication, and acoustic links. Electromagnetic communication is not suitable for underwater communication because of its limitation of higher attenuation resulting in higher absorption loss due to the conductivity of water. Similarly, optic signals are not supported by the aquatic environment because of the requirement of the direct line of sight between the sender and receiver [1]. The most suitable medium for underwater communication is the acoustic channel; however, there also exist several challenges, e.g., limited battery power, fading, limited bandwidth, high error rate [2].

UWSNs communication’s performance is gauged over several performance metrics including energy tax, Packet Delivery Ratio (PDR), End-to-End Delay (E2ED), and Accumulated Propagation Distance (APD). These parameters are affected by various factors, e.g., void holes, energy holes, duplication of packets. Energy optimization is one of the main requirements for increasing the lifetime of a battery and overcoming other challenges such as void/energy holes, packet duplication, etc. The void/energy holes are created when a sender node cannot find other appropriate nodes for forwarding the received data packets due to the lack of information about the surroundings. Similarly, the duplication of packets happens when the source transmits data in the Omni direction which is received by all neighbor nodes for further transmission. Due to such data duplication, a significant fraction of energy is used in the transmission phase resulting in reduced network lifetime. The battery replacement of an underwater node after deployment is not feasible. Several routing protocols and schemes are proposed in the literature for efficient communication in UWSNs; however, each of them has its overheads as discussed in Section 2: Related Work. In particular, high energy consumption is still an important concern, and therefore, in this research work, we propose a new protocol, called “An Efficient Routing Protocol based on Master–Slave Architecture for Underwater Wireless Sensor Network (ERPMSA-UWSN)” focusing on energy optimization.

Following are the main contributions of this research work:

- Our proposed master–slave architecture helps to achieve lower energy consumption by limiting the broadcasting of packets to a specific region (set of nodes).
- We avoid packet duplication due to the suppression of nodes and the lack of multiple paths for transmission.
- We perform extensive simulation of our proposed protocol in various scenarios and demonstrate improvements in Packet Delivery Ratio (PDR) and energy tax.

The rest of the manuscript is organized as follows: Section 2 contains a detailed discussion on the previous work regarding UWSN-based routing protocols. Section 3 identifies the problem statement that we aim to address in this research work. The proposed system model is presented in Section 4. Section 5 delineates the experimental setup and simulation results, performance comparison, and analysis of our proposed approaches. The paper is concluded in Section 6.

## 2. Related Work

This section discusses several existing protocols in the domain of Underwater Sensor Networks (UWSNs). In addition, the differences in routing protocols used in UWSNs are also highlighted. Energy Efficiency in UWSNs is one of the major concerns highlighted in the literature.

The existing UWSNs protocols can be divided into the following two categories: (i) localization-based protocols, (ii) localization-free protocols [3]. Table 1 lists the protocols in these categories along with their advantages and disadvantages. The detailed discussion of protocols in each category is given below.

### 2.1. Localization-Based Routing Protocols

These protocols need the location information in two or three-dimensional distribution of the node for calculating the optimal routes for transmission of the data from the sender to receiver end of the network. Such information of coordinates is required for the calculation of the distance between the two nodes and routing trajectories of the routing protocols. The following protocols are classified into this category based on their parameters and the solutions they propose.

- VBF: The Vector-Based Forwarding (VBF) [4] protocol uses a virtual pipe core in a virtual vector for data forwarding. The virtual vector extends from source to destination and chooses only the nodes lying in the pipe. The protocol uses redundant and different routes for stability in terms of node movement from source to destination. However, by choosing the forwarders within the pipe repeatedly, the energy usage is not optimized. Furthermore, the network lifetime is high due to imbalanced energy usage.
- HH-VBF: To increase the efficiency of VBF, another protocol called Hop-by-Hop Vector-Based Forwarding (HH-VBF) has been proposed in [5]. In this protocol, every node generates a virtual pipe that changes the vector path by the node position. In contrast to VBF, energy consumption is balanced in this protocol. However, the HH virtual pipe approach creates significant contact overheads compared to VBF and lacks a mechanism to avoid the void space.
- AHH-VBF: The Adaptive Hop-by-Hop Routing Protocol (AHH-VBF) [6] is another attempt to improve the VBF and HH-VBF protocols. It uses variable transmission ranges to save network resources. The transmission range of AHH-VBF varies by distance from the farthest carrier and improves the network lifetime due to the adjustment of the holding time. However, this protocol is limited to select the forwarder by ignoring any residual energy which results in replication packages and unbalanced energy usage.
- ANU-AHH-VBF: In [7], the author suggests another location-based protocol called Avoiding Void Node with Adaptive Hop-by-Hop Vector-Based Forwarding (AVN-AHH-VBF) that uses a virtual routing pipeline with the configured radius for data analysis. In this protocol, when a node receives a packet, the distance from the forwarder is observed in the predefined range. The AVN-AHH-VBF uses the concept of holding time to reduce unwanted broadcasts. The PDR is improved because the packet collision is minimized. However, the performance concerning end-to-end delay is not improved. Therefore, by using residual energy, the technique could not achieve the balancing of energy consumption among nodes.
- ESEVBF: In [8], the authors proposed Energy Scaled and Expanded Vector-Based Forwarding (ESEVBF) protocol to offset energy consumption. The selection is based on the residual energy of the forwarder nodes. It measures and increases the time difference of holding with the residual PFN energy. While lowering the end-to-end delay, the selection process achieves reduced packets’ duplication and network energy consumption. The routing protocol demonstrates little changes in Packet Delivery Rate (PDR), as the forwarding node suppresses vast amounts of nodes with a slight variation in the residual energy, inside the potential forwarding region. If a communication void hole happens, VBF fails to react.
- NEFP: The Novel Efficient Forwarding Protocol (NEFP) [9] is also a localization-based routing protocol. It has three main tasks, i.e., forwarding/routing zones to avoid unimportant forwarding using Markov chain to measure the forwarding probability of the packets in different network topologies and the calculation of holding time to avoid packet duplication and Collison. However, this protocol does not perform well in sparse conditions because of the less probability of finding the next forwarder in the forwarding zone.
- TC-VBF: To address the issue of node’s communication in sparse environment, the Topology Control VBF (TC-VBF) [10] was introduced. It performs the selection of the forwarder similar to the technique of VBF but with the addition of network density consideration. However, eventually, this also faces the same issue of energy holes and void holes such as the VBF in the sparse network due to the death of the forwarder nodes.
- MEES: In this protocol, the two mobile sinks are chosen which are far away from each other [11]. Data collection from the nodes is performed via predefined linear paths. The experimental results demonstrate the improvement of network throughput and lifetime as well as energy consumption.
- DTMR: To reduce energy consumption in terms of signaling power and transmission delay, the authors in [12] proposed the Direction Transmission or Mobile Relay (DTMR) protocol where a source node can send data through a mobile relay node or directly to the destination node; however, there is no guarantee for the reliable communication of data to the destination because of the harsh underwater environment.
- FVBF: The Fuzzy logic-based Vector-based Forwarding protocol (FVBF) [13] is another attempt to improve VBF. It considers the valid distance, projection, and energy of a node for the selection of the next forwarder. Valid distance determines the closeness of a node to sink while projection determines its closeness to the routing pipe.

### 2.2. Localization-Free Routing Protocols

We mainly focus on the localization-free routing protocols, also called depth-based routing protocols, as they are more closely related to our proposed work. The routing schemes in such protocols use the depth information to evaluate a communication route between the source and destination. This information is being processed iteratively on each hop until it finds the destination. Following are the well-known localization-free (depth-based) protocols in the literature.

- DBR: Depth-based Routing (DBR) [14] is the base protocol that played a vital role in the transformation from localization-based to depth-based routing in UWSNs. DBR is an approach based on the greedy algorithm focusing on sending packets from source to sink nodes. It is a single-hop scheme that uses the next node for the data packet transmission. The nodes are randomly deployed and have the characteristic of mobility due to the ocean nature, therefore, resulting in the void hole problem. This protocol uses the depth information to select the next transmission forwarder, and the source node transmits the data in its premises, while every other node within the range of the network receives the packet. Based on the depth difference information, the surrounding nodes are divided into PFNs and suppressed nodes. Next, the nodes occurring in the lower hemisphere drop the packet while the nodes in the upper hemisphere (i.e., PFNs) determine the holding time using the local depth information, and set the timer to hold the data packet. During this holding period, if a node does not obtain a copied version of the captured data packet, it transmits the packet after the timer expiration. On the other hand, the packet is dropped. In this protocol, the sender makes decisions based on one-hop depth and remaining information, resulting in a lower rate of packet delivery as well as lower energy efficiency, and eventually leading to the occurrence of void and energy holes.
- DSRP: Akanksha et al. [15] presented the Distributed Delay-Sensitive Routing Protocol (DSRP) to address the mobility of nodes in a network. In this model, when a node reaches its destination, it stops there for a moment and is then shifted to the new destination by keeping account of the expected traffic, chosen paths, and localization of the nodes. The movement speed of the node is kept between maximum and minimum values. This high mobility results in high PDR with the cost of high energy consumption and end-to-end delay, as compared to low mobility and no mobility models.
- ODBR: The Optimized Depth-Based Routing (ODBR) protocol [16] targets the energy balancing approach where it assigns the energy to nodes based on their depths. To prolong the network lifetime and balance the energy consumption, the nodes with smaller depths are assigned a high amount of energy while the nodes with higher depths get low energy. This approach is not applicable in a deep-water environment because the nodes at the highest depth get low energy and die quickly. However, it works well in shallow water because each node in the network gets the required amount of energy to transmit data due to its less depth.
- EBECRP: To reduce multi-hopping, the authors in [17] presented an Energy-efficient and Balanced Energy Consumption Cluster-based Routing Protocol (EBECRP) which divide the network into sectors where each sector consists of a cluster head capable of collecting data from its neighbor nodes. The nodes first send data either directly to the sink or the cluster head and then the sink collects data from the cluster head. This results in low energy consumption due to optimized routing but high packet loss because of the early death of the cluster head due to energy reuse.
- Hydrocast: The Hydrocast protocol [18] proposed a way to use water pressure as a driving force to forward a data packet to the sink which reduces energy balancing and economize the node energy. To ensure the successful packet delivery to the destination, a dead-end recovery method is proposed where a node hands over its data packet to the neighbor with greater energy. Therefore, it does not perform well in sparse conditions because it cannot find neighbors.
- WDFAD-DBR: The WDFAD-DBR protocol [19] is an improved version of DBR that aims to make the routing decisions more intelligent by providing two-hop information. Unlike the DBR, this scheme is based on a two-hop model, where the depth information of the next node’s and expected hop is used to calculate the holding time. In addition, the forwarding area is split into three parts, i.e., one main region and two secondary regions for forwarding, so that the high priority nodes can suppress low priority nodes. WDFAD-DBR try to avoid the void hole in advance, as it considers the depth of the expected next-hop neighbor in addition to the current forwarding neighbor. WDFAD-DBR achieves a good packet delivery ratio in a sparse network; however, the forwarding strategy fails to balance network traffic and lower depth nodes are penalized which leads to the early death of the network. Because of the stretching of holding time differences among neighboring nodes, the duplicate packets are not reduced which further results in high energy consumption.
- DOW-PR: The Dolphin and Whale Pod Routing protocol (DOW-PR) is introduced in [20] to achieve better performance for WDFAD-DBR. This protocol adopted the weighting depth difference for the forwarder selection, with some additional selection parameters i.e., PFN number, hop count, and the number of nodes suppressed as well as node residual energy. This hashed out the problem of void holes up to some extent by using the path from the suppressed region in the absence of nodes in the PFN region. The DOW-PR involves two processes i.e., (i) Whale Pod routing (ii) Dolphin Pod routing. In Dolphin Pod routing, all the sink nodes are installed on the water’s surface, whereas only a single sink is placed inside water in the case of Whale Pod routing. Additionally, the power range is divided into power levels for better use of the node power and traffic control using the hop-count mechanism. The experimental results (simulation-based) showed significant performance in terms of higher packet delivery rate, lower APD, smaller energy tax, and better network life.
- EEPDBR: EEPDBR [21] algorithm is used to design an improved probabilistic DBR algorithm for underwater data collection and reporting to the surface receivers by taking node’s residual energy, depth information, and the number of PFNs within the 2-hop neighborhood. This technique outperforms in terms of PDR, energy efficiency, and low average delivery time.
- EBH-DBR: EBH-DBR [22] uses depth, residual energy of the node, and reliability of the link to select the next relay node for data forwarding. In this scheme, the network is divided into slices of the same width to balance energy consumption as well as to control the hop count of the sensor nodes forwarding the data. This technique works well in terms of network prolonging, energy balancing, throughput, and transmission loss.
- EECMR: Energy-Efficient Clustering Multi-hop Routing (EECMR) protocol [23] aims to balance energy consumption of the data transmitting nodes and prolong their life. In this approach, the network is divided into multiple layers concerning the depth level. Data is being forwarded to the sink using a multi-hop mechanism by the cluster head selected based on the depth and residual energy of the node. The cluster head then aggregates the data packets of all cluster members and sends them to the upper layer of the sink node. This protocol is effective in terms of network lifetime and energy consumption.

Several other depth-based routing protocols are also proposed for mitigating underwater network challenges, e.g., OMR [24], QERP [25], EECOR [26] and RRSS [27], UMDR [28], Stochastic modeling of depth-based routing [29], PCR [30], Energy-Efficient and Void Avoidance Routing [31].

The Cross-layer Mobile Data gathering (CLMD) [32] protocol is also proposed to improve the network performance by creating interaction between MAC layer and routing layers. An Autonomous Underwater Vehicle (AUV) is used to visit each cluster collecting data from the neighborhood. The communications are managed through a distributed cross-layer solution. To prolong the network lifetime, the cluster heads are replaced with other cluster heads at the end of each operational phase. This protocol outperforms in terms of energy optimization, network lifetime, and PDR of the network. Similarly, Gul et al. [33] presented an Energy-Efficient Regional Base Cooperative Routing protocol (EERBCR) with sink mobility by dividing the network field into 12 regions. Four mobile sinks are deployed at equal distance in the randomly deployed 100 nodes. Each sink travels on a predefined route to cover all 3 regions while all other nodes are in sleep mode. A sink enters the region and broadcasts a hello message in its range to activate the sleeping nodes. At the time of departure, it leaves another message to put them back to an indolent state. This protocol performs well in terms of energy optimization and prolonging the network lifetime.

In addition to the above studies, some recent studies in UWSNs include a comprehensive survey [34] describing the requirements, taxonomy, recent advances, and open research challenges in the field of Underwater Sensor Networks. Similarly, energy optimization of underwater sensor network is explored recently through depth-based routing [35], clustering scheme [36], machine learning technique [37], and reinforcement learning [38]. A comparative study on the applications, deployment, and routing techniques in UWSNs is also carried out in [39].

The most closely related work with our study is WDFAD-DBR [19] which uses the two-hop methodology and considers depth as well as residual energy of the nodes, PFN of the nodes, and locality of the node for the next forwarder (master node) selection. Such parameters significantly contribute to addressing the problem of void and energy holes in the network. Additionally, Data packet reception is limited to only master nodes, which contribute to energy efficiency and data packet collision avoidance. In addition to these features of WDFAD-DBR, our proposed protocol also uses adaptive transmission power adjustment based on the locality of the next forwarder to further reduce the energy consumption. Other features and working of the protocol are explained in the later sections.

All the UWSN routing protocols, discussed in this section, are listed in Table 1 along with their advantages and disadvantages.

## 3. Problem Statement

In this research work, we aim to address the problem of high energy consumption of data packet transmission in UWSNs. The two existing protocols i.e., DBR and WDFAD-DBR transmit data packets by broadcasting them across all over the Potential Forwarding Zone (PFZ), and all neighbor nodes receive the transmitted packet. Next, the node with higher priority (smaller holding time) transmits the received packet further while the remaining nodes drop it. In both schemes, there is a wastage of energy in the form of redundant data packet transmission and unnecessary reception of data packets by suppressed nodes. The data packet has a larger size than the other packets (e.g., Neighbor request and Acknowledgment), due to which the nodes consume more energy for transmission.

**Figure 1:**
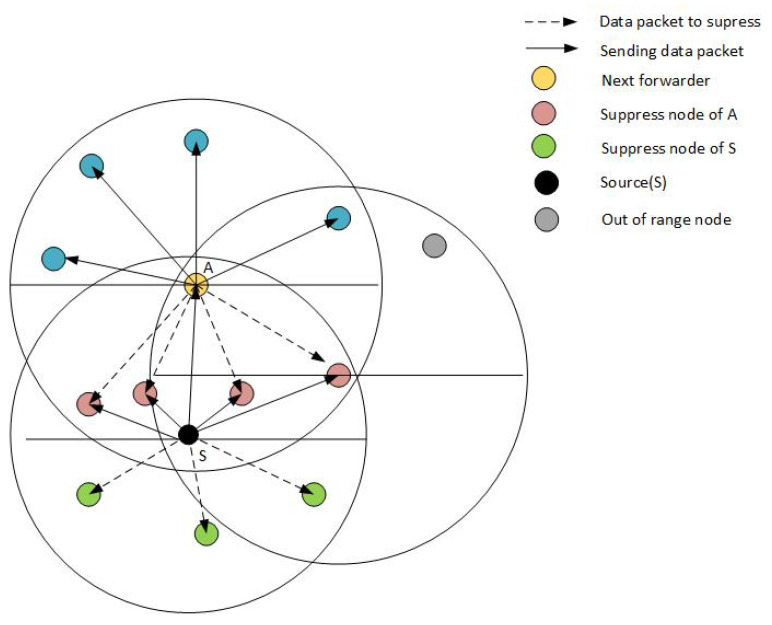
Selection of next PFN in WDFAD.

WDFAD-DBR is an improved version of DBR and is the basis of our work, therefore, we explain the data packet transmission in this protocol with the help of Figure 1. Node S is the source node that gathers information about the neighbor nodes by broadcasting the Neighbor request and gets the Acknowledgment packet in response. Next, the S node calculates and assigns the holding time of each node using a fitness function and starts its data packet transmission. As depicted in the figure, node A has a smaller holding time and starts data packet transmission while the other (four) nodes, with higher holding time than A, wait for their turns. Since these suppressed nodes are also in the transmission range of A, they receive the same packet back as node A transmits it. Although these nodes will not become part of further transmission, energy is still wasted due to the reception of packets. All other nodes outside the region of A (suppressed nodes of S) do not receive the data packet from node A and therefore start their transmission resulting in data packet duplication and high energy consumption. For resolving this problem, we propose the ERPMSA-UWSN protocol that mainly focuses on reducing high energy consumption in UWSNs.

## 4. System Model

In this section, we define some basic terminologies, network architecture, and our proposed network topology.

### 4.1. Basic Terminologies

Following are the basic terminologies used in our proposed protocol ERPMSA-UWSN protocol.

- Master nodes: Master nodes are those nodes that fulfill the requirements of the Master Selector function (MSF) and are eligible for the forwarding data packets.
- Slave nodes: Nodes other than masters are slave nodes and are not eligible for forwarding data packets.
- Anchor nodes: Nodes which are statically bounded at the bottom of the water, also known as data collectors or source nodes.
- Relay nodes: Nodes which are suspended in water and work as 3rd party between anchor nodes and sink nodes.
- Sink nodes: Sink nodes are the receiver/destination nodes and they float on the surface of the water. Their responsibility is to collect data packets from sensor nodes and forward them to base stations through a radio link.
- Transmission range of node S: This refers to the omnidirectional distance from source node S (Xs, Ys, Zs) which currently forwards the packet p until and unless the packet is transmitted.
- Void hole: A state where the forwarding node cannot find any other node to further forward a packet.
- Energy hole: A scenario where the forwarding node lacks the required energy for forwarding a data packet.
- Eligible Neighbors (*En_i_*) of Node i: Nodes spanning within the transmission range of a given node *i*. Let’s suppose *N* represents a set of nodes in a network
  (1) $$N=n_{1},n_{2},n_{3},.......,n_{k}$$
  Therefore, the eligible neighbors of a Node *i* can be illustrated as Eni⊆N
  (2) $$En_{i}=\left\{j\in N\wedge Dist_{j}^{i}\leq R_{r}^{i}\right\}$$
  where (*Dist_j_^i^*) is the Euclidean distance between node *i* ($x_{i},y_{i},z_{i}$) and node *j* ($x_{j},y_{j},z_{j}$) in a 3-dimensional Euclidean space:
  (3) $$Dist_{j}^{i}=\sqrt{{\left(x_{i}-x_{j}\right)}^{2}+{\left(y_{i}-y_{j}\right)}^{2}+{\left(z_{i}-z_{j}\right)}^{2}}$$
- Potential Forwarders (*Pf_i_*) for a Node *i*: Nodes lying in the transmission range *R_r_^s^* and with their depth (*d_j_*) less than the depth (*d_i_*) form the potential forwarders for a node i, as given below:
  $$Pf_{i}\subseteq En_{i}$$
  where
  $$Pf_{i}=\left\{j\in En_{i}\wedge d_{i}<d\right\}$$
- Potential Forwarding Zone (PFZ): The Potential Forwarding Zone (PFZ) creates a hemispherical zone, where the radius is similar to *R_r_^s^* and the distance to the sink for each point of PFZ is lower than that of the source node. The PFZ is the sub-region of *R_r_^s^* for node S and the nodes residing in the zone are Potential Forwarder Nodes (PFNs), i.e., the subsequent forwarders for the packet p.

All abbreviations/symbols used in this manuscript are listed in Table 2.

### 4.2. Network Architecture of ERPMSA-UWSN

In this section, the network architecture of our proposed protocol is presented. We describe the masters-slave technique of forwarding packets that aims to achieve low energy consumption.

**Figure 2:**
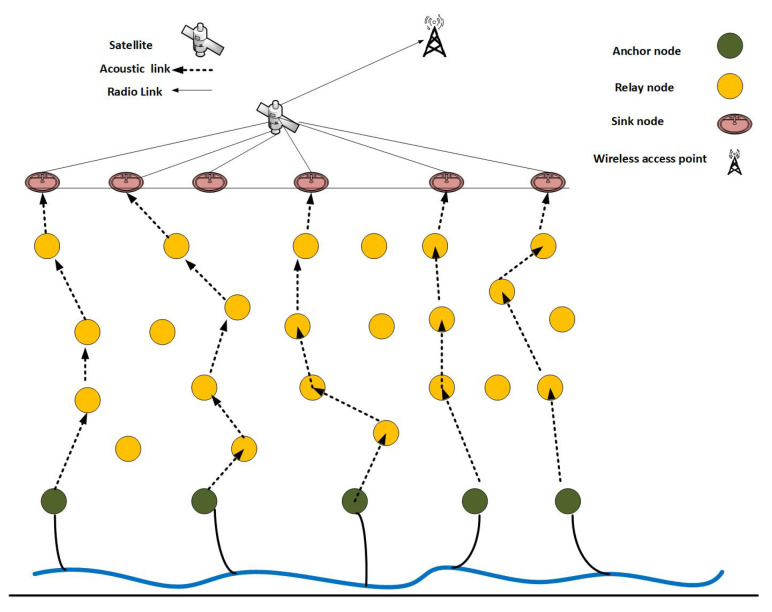
Network architecture.

The network architecture of ERPMSA-UWSN is constituted of anchor nodes, relay nodes, and sink nodes as depicted in Figure 2. Sink nodes float on the surface bearing radio and acoustic modems to communicate with each other and relay nodes, respectively. These nodes can receive signals from water and perform the transmission/reception in the external environment without any boundaries. Relay nodes are deployed at different levels of depth, act as the 3rd party between the transmitters (anchor nodes) and receivers (sink nodes), and carry the transmitted data towards the receiver. Anchor nodes are tethered alongside seabed and can move with water currents or with other environmental disruption. Their main task is to gather environmental data and pass it to the sink nodes via relay nodes. The centralized stations are the sink nodes that enable communication with other sink nodes as well as relay nodes. The reception of the data packet on any of the sink nodes is measured as a successful packet delivery.

### 4.3. Impact of Acoustic Signal Velocity in the Underwater Environment

The velocity of the sound is affected by various factors including temperature, pressure, and the salinity level of the seawater. Mathematically, it can be elaborated as follows [40]:

$$\begin{array}{c} c=1446.961+4.591T-5.305\times 10^{-2}T^{2}+2.374\times 10^{-2}T^{3}\\ +1.340\left(S-35\right)+1.63\times 10^{-1}D+1.6750\times 10^{-7}D^{2}-\\ 1.025\times 10^{-2}T\left(S-35\right)-7.1390\times 10^{-13}TD^{3} \end{array}$$

where *c* is the velocity of the sound signal measured in m/s, *T* shows the temperature whose unit is Celsius, *S* indicates the salinity of water with the unit of Part Per Thousand i.e., PPT and *D* pertains to the depth measured in meters. The above equation qualifies for 0 C ≤T≤30 C, 30≤S≤40 PPT, 0≤D≤8000 m.

### 4.4. The Energy Propagation Model

The attenuation of the acoustic channel in UWSNs over depth *d* is explained by the following formula [41]:   

(4) 10∗logA(d,f)=k.10∗logd+d.10∗logα(f)

In the above equation, the right side occupies two terms, i.e., the spreading loss and absorption loss, respectively, where *k* is a constant whose values are 1, 1.5, and 2, and constitutes the geometry of propagation. Varying the values of *k* affects the geometry accordingly. In shallow water, the propagation geometry is cylindrical if the value of *k* = 1, spherical in deep water if *k* = 2. Similarly, for *k* = 1.5, the geometry of the propagation is practically spread, where α(f) represents the absorption coefficient.

The following expression can be used to find the underwater noise.

(5) $$N\left(f\right)=N_{t}\left(f\right)+N_{s}\left(f\right)+N_{w}\left(f\right)+N_{th}\left(f\right)$$

where $N_{t}\left(f\right),N_{s}\left(f\right),N_{w}\left(f\right)$ and $N_{th}\left(f\right)$ represent the turbulence noise, shipping noise, wave noise, and thermal noise, respectively.

### 4.5. Influence of Voice Signal Reflection/Refraction in an Underwater Environment

The channel geometry and its properties of reflection and refraction impact the impulse response of an acoustic channel. These characteristics determine the total count of the major paths and their relative strengths as well as delays. After discarding the echoes which undergo multiple reflections from almost infinite signal echoes, only a few significant paths are left. The total multi-path spread is governed by the longest path delay, which is for the tuning of tens of milliseconds. These types of values are reported in the shallow water experiments [42]. The total multi-path spread is far greater than the dispersion of the individual paths. Therefore, it can be ignored for the systems whose channel cutoff is greater than most of the frequencies. For systems currently in use, this is typically the case. We ignore the reflection phenomena for both ERPMSA-UWSN and WDFAD-DBR, in our simulations, for the sake of fair analysis.

### 4.6. Packet Types in ERPMSA-UWSN

Following are the four types of packets in ERPMSA-UWSN (i.e., NR, DATA, ACK, and CTRL).

- Neighbor Request Packet (NR): The source node uses NR to get information about the neighbor forwarders. The format of the NR packet is NR (TYPO, SID, D, VA), where TYPO is the two-bit number indicating the type of the packet. For NR, the value of TYPO = 00. SID is the ID of the source node and D is the depth of the source node.
- Acknowledgment Packet (ACK): ACK packet is a reply from the neighbor node in the response of NR, containing full information of its sender. The format of the ACK is ACK (TYPO, SID, D, E), where the value of TYPO for ACK is “01”, SID is the ID of that neighbor node, D is the depth and E is the energy of the ACK packet’s sender node. We store these values in a separate table called MTAB, in descending order corresponding to their IDs. In the table, the MSF value of the first index node is highest among all nodes and other indices are occupied accordingly.
- Control Packet (CTRL): CTRL packet is used to invoke the masters for the forwarding procedure. By awakening the masters, it automatically turns slave nodes into the indolent state. The format is CTRL (TYPO, SID, PTR1, PTR2, D) where TYPO for this packet is “10”, SID is the ID of the sender node, PTR1 and PTR2 are pointers to the array that contain IDs of the three descending indexed Masters of PFZ1 and PFZ2 respectively, while D is the depth of the sender node.
- Data Packet (DATA): DATA packet contains the real data with the payload and header. The format of the DATA packet is DATA (TYPO, SID, DID, D), where TYPO value is “11”, SID represents the source node ID, DID indicates the destination node, while D represents the depth of the sender node.

#### 4.6.1. Division of Potential Forwarding Zone (PFZ) into PFN levels

**Figure 3:**
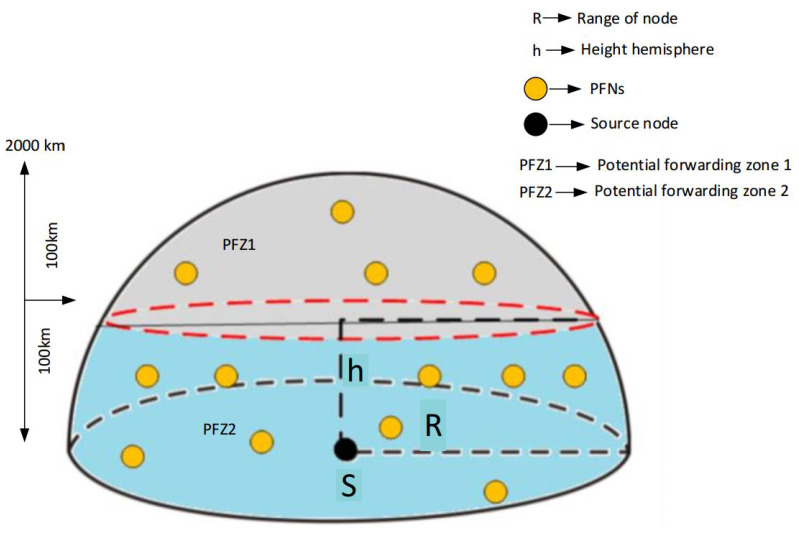
Region division of PFZ1 and PFZ2.

In this scheme, the division of PFZ into different PFZ levels is based on the depth difference of the source node and the PFN zone. To achieve optimal transmission of the data packet, we tend to access the master node closer to the sink. For this purpose, PFZ is divided into two equal regions based on the depth difference as shown in Figure 3. As the transmission range is 2000 m, the region where the depth difference is between 1001 m to 2000 m is considered to be PFZ1, while from 1 m to 1000 m, it is considered to be PFZ2. This 3D division results in a truncated hemisphere with an upper cap (PFZ1).

#### 4.6.2. Node Density in PFZ Levels

The node density in a certain place indicates less probability of void hole occurrence and the chances of getting the best nodes for forwarding. This results in two regions, i.e., the region with dense nodes but with more distance from a sink (PFZ1), and the region with less depth but with a sparse setup (PFZ2). The first choice PFZ1 can result in attaining high PDR on the cost of high APD and high energy use. On the other hand, in the second choice (PFZ2), low energy consumption and high PDR can be achieved because our proposed system topology outperforms in a sparse environment in terms of PDR and energy [43].

In ERPMSA-UWSN, the next forwarder (Master) is selected only if it has PFNs in its PFZs and has enough energy for transmission. Consequently, this minimizes the probability of void holes and energy holes in a less dense region. Therefore, to gain the desired output we prefer the first choice i.e., prioritizing PFZ1 over PFZ2 for master selection.

**Figure 4:**
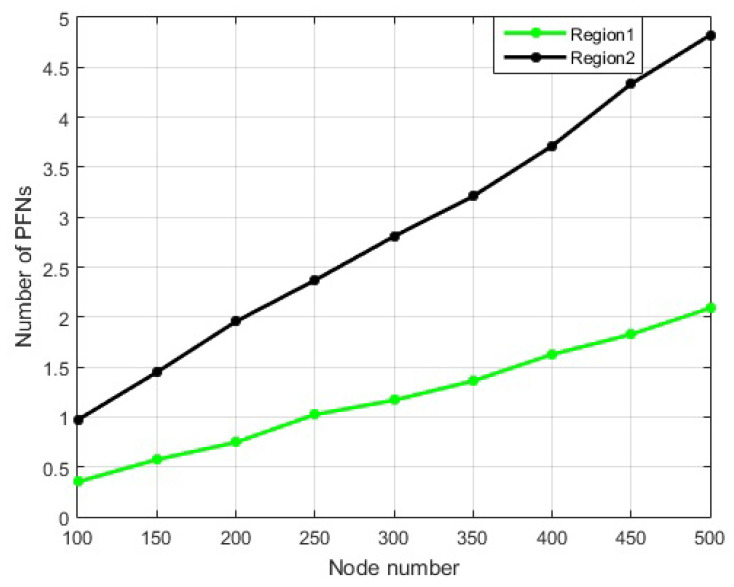
Number of Nodes in Each Region.

We established the knowledge about node density in PFZ1 and PFZ2 based on the theoretical analysis that we carried out with the volumetric information and then justified it by experimental results. By applying Equation (6), we calculated the volume for the upper cap of the hemisphere (PFZ1) using parameters h = 1000 m and r = 2000 m. Similarly, for the lower partial hemisphere, we used Equation (7), for the calculation of volume. Comparing the volumes, we found out that PFZ2 has more capacity than PFZ1, indicating that the probability of nodes is higher in PFZ2 as shown in Figure 4.

(6) $$V_{upper}=\frac{1}{3}\pi \left(3hr^{2}-h^{3}\right)$$

(7) $$V_{lower}=\frac{1}{3}\pi h\left(3r^{2}-h^{2}\right)$$

### 4.7. Distance-Based Adaptive Transmission Methods

In prior approaches, the use of transmission energy was not economical. In our proposed technique, we produced an idea to efficiently limit energy usage by ranging transmission energy on different depth levels. This approach mainly restricts the transmission energy to beneficial usage. We use adaptive energy levels that are devised, based on the distance between the source node and master node. In previous practices, two types of mechanisms were followed. First, the source node sends neighbor requests in its range with power P, and in response, the neighboring PFNs send Acknowledgments back to the source containing all required information. Secondly, when the source node receives Acknowledgments from its neighbors, it compares their depth parameter, broadcasts data packet with power P to all neighbors while setting the awaiting timer based on their depth information. At this stage, the node closer to the source might need less transmission energy from the source. However, it is transmitted towards the neighbor with the same power needed to transmit over R which is unacceptable in a scenario of optimizing energy consumption. To address this issue, we restrict additional energy costs that the network bear for data transmission in the close neighborhood of the source. For this purpose, we use the technique where the transmission energy assignment is based on the distance between the source node and master node. The larger distance between the source and master nodes means greater transmission energy and vice versa.

### 4.8. Distribution of PFNs into Masters and Slaves

**Figure 5:**
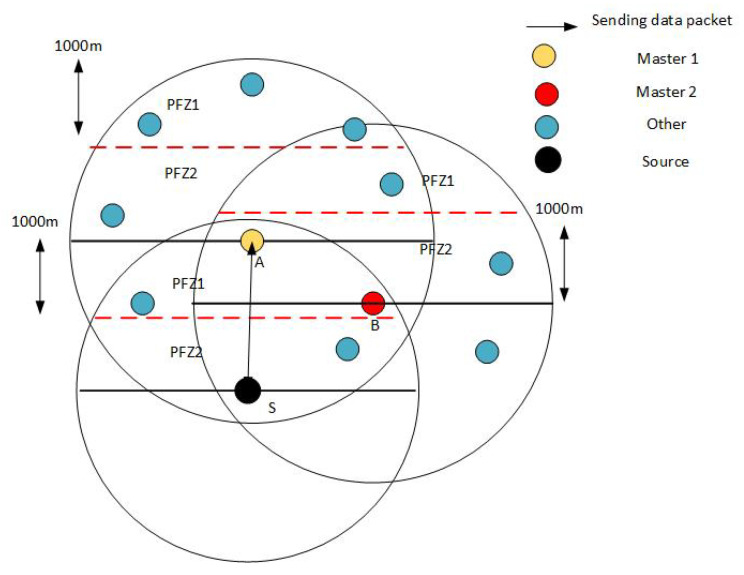
Master and Slave division.

The distribution of master and slave nodes is based on the values of the Master Selector Function (MSF). For the top 3 values of MSF, we rank the nodes as masters and the rest as slaves for that region. This master–slave race exists in both PFZ1 and PFZ2 regions.

(8) $$MSF=\frac{\left(P1+P2\right)*E_{n}}{Z}$$

where *E_n_* is the normalized form of that PFN’s energy for whom we are finding the MSF. The parameter *Z* is the normalized depth, whose value ranges between 0 and 1 of that PFN. The parameters *P1* and *P2* are priority factors, dependent upon the population of PFNs in PFZ1 and PFZ2 regions of the next master respectively, while their values range between 1.0 and 0.5. These values are only applicable if the number of PFNs in both the regions (PFZ1 and PFZ2) is equal to or greater than 1. To find the next forwarder (master) closer to the sink, we considered these priority values. As discussed above, the forwarder will have a higher value of MSF. The value that contributes more in MSF is *P1* = 1 as compared to *P2* = 0.5 which clearly shows that the region PFZ1 gets the higher probability and hence the desired forwarder. Complete elaboration of this process is depicted in Figure 5.

Our proposed approach is based on the master–slave architecture. The selection of master nodes is carried out via the defined Algorithm 1 (Master Selection Algorithm). The selection is based on the information of the neighboring nodes, stored in a neighboring table. Once a master node is selected, its information is stored in the master nodes table, which can be further used for selection at run time. The master node information of each node is stored in the mentioned master table. Before broadcasting a data packet to the surrounding, a control packet is sent to the neighborhood, to activate all master nodes and deactivate slave nodes. However, since it is a challenging task to accomplish at run time, we devised a formula called Master Selection Function (MSF). The mentioned formula filters out the best nodes among the neighbor nodes based on the energy, small depth difference, and number of PFNs. The resulted values from MSF are stored in an array, while the top 3 values from the array are stored in the master table. We assume that each transmitting node has the information of its neighbors as well as master nodes, as periodic requests are sent for updating the neighbor and master tables. These functions are achieved via control packets, which are used to broadcast control signals across the network and to enable or disable other nodes.
**Algorithm 1:** Masters selection algorithm
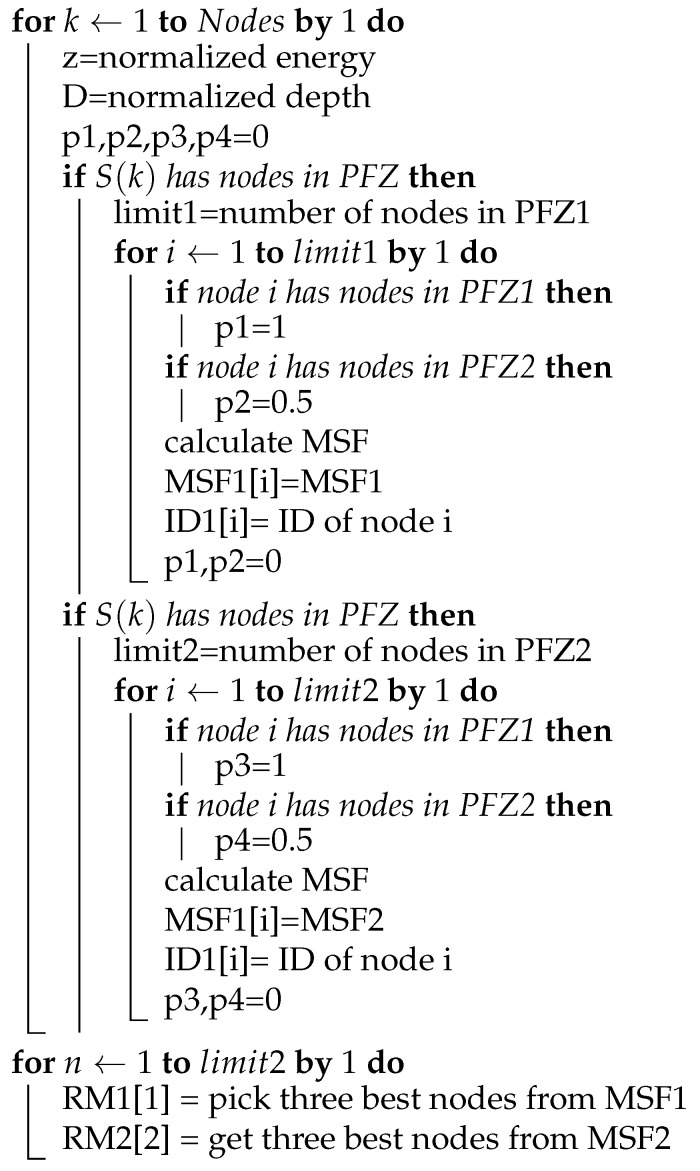


### 4.9. Master-Range Extension to End Data Packet Duplication

**Figure 6:**
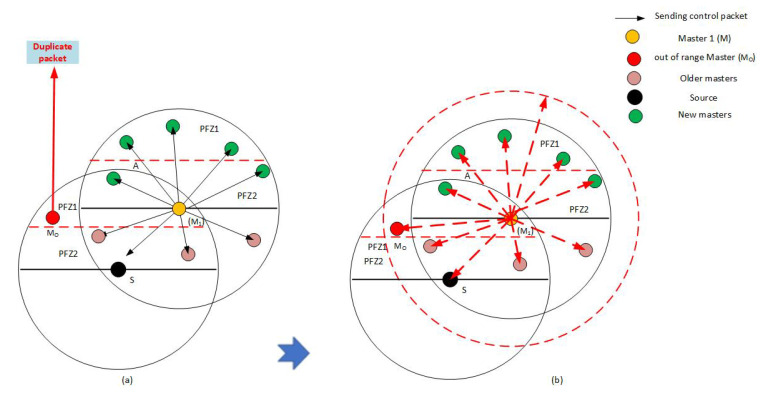
(**a**) Packet duplication (**b**) Master-Range extension to end data packet duplication.

In the transmission of the data packet, in rare cases, some of the master nodes cause data packet duplication. This happens when some masters are situated beyond the boundary of the master responsible for forwarding the data packet. Consequently, they cannot receive a control packet from it and, in response, after the expiry of their holding time, consider themselves eligible for forwarding. To deal with this issue, we change the range of the control packets adaptively with the position of masters which are out of the privileged master’s range as shown in Figure 6a. When master A sends a control packet to suppress the slave nodes of the next-hop and master nodes of the previous hop, master M_O_ will not receive the control packet. Hence, after the holding time elapses, it will generate a duplicate data packet. To address this problem, node A checks the presence of all master nodes in its range before sending the control packet. If it finds any of them outside the range, it finds the distance between that node and itself using Equation (9), and re-adjusts its control packet energy for that new range as shown in Figure 6b. If it broadcasts the control packet, it will suppress node M_O_ from generating a duplicate packet. This happens quite rarely in our topology because the selection process considers that the masters should be selected only when they are close to each other. Also, such deviation from MSF is possible in a sparse network.

The master-range extension is based on the distance between the master nodes. This distance is calculated using the following Equation (4):

$$Dist_{j}^{i}=\sqrt{{\left(x_{i}-x_{j}\right)}^{2}+{\left(y_{i}-y_{j}\right)}^{2}+{\left(z_{i}-z_{j}\right)}^{2}}$$

in case the node is falling outside the range of the forwarding master node, we use this distance to calculate the extended region of the master node for suppressing the duplication of the data packet. Such calculation can be expressed using the following equation:

(9) $$R_{ext}=Dist_{M_{O}}^{M_{1}}-R$$

where *R_ext_* represents the extra range (master-range extension), $Dist_{M_{O}}^{M_{1}}$ is the distance between first master (*M*_1_) and the other master which is the out-of-range (*M_O_*) node, while R is the transmission range.

### 4.10. Data Packet Forwarding Mechanism in ERPMSA-UWSN

The following steps are taken to transmit the data packet towards its destination point. First, when a node becomes a source node it will broadcast neighbor request (NR) in its range and gets the Acknowledgment (ACK) in response from neighbor nodes.

**Figure 7:**
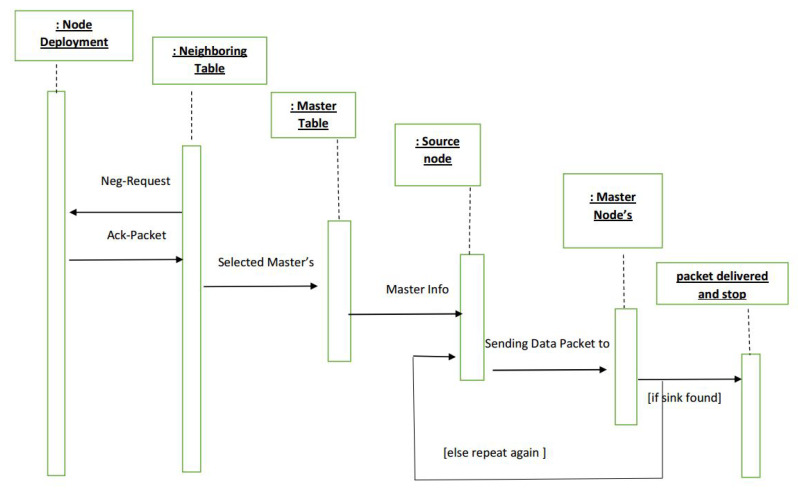
Sequence Diagram of ERPMSA-UWSN.

- After receiving the ACK packet, the source node updates its neighbor table and sorts out the master and slaves.
- The source node sends a Control packet in its surroundings, containing the IDs of Masters of each PFN region, which turn the slave nodes to the idle state while keeping the masters active.
- The data packet is sent which is only received by the master nodes while the holding time is assigned based on their priority. In this way, masters nodes of the region PFZ1 get a higher priority than the region PFZ2.
- Before forwarding data, master must check for the energy hole scenario. In case of an energy hole, the 2nd Master takes charge and forwards the data packet. If the 2nd Master also encounters the same situation, it hands over the responsibility to its subordinate master, i.e., the 3rd master. If it does not find a way out, the packet is dropped. This increases the chances of the data packet getting its destination most probably.
- If there is no energy hole then the master beating the clock first will forward the data to the forwarder in its range in the same fashion, while the other master drops the packet after hearing the control packet from the forwarding master.
  The whole procedure is well elaborated in the sequence diagram in Figure 7.

### 4.11. Holding Time and Activation Time Calculation

When a node transmits data, it must allocate two types of timer slots to the receiving masters i.e., activation time and holding time. Activation time and holding times are associated with the control packet and data packet, respectively. The control packet is released after completion of Activation time. Similarly, the data packet broadcasts right after the last tick of the holding time clock. These timers are allocated based on the MSF values of master nodes. Master node with small-time span is the nodes, whose depth is low compared to others and when its next-hop master lies in its PFZ1, while having more energy than others.

Furthermore, the timer is separately allocated to the three of the masters in that PFN region. If in case the source node does not find any master in PFZ1, forwarding will be formed in PFZ2, and their timer allocation will be of different magnitude.

#### 4.11.1. Activation Time (AT) calculation

Activation time for the master node is the time before spreading the control packet in its neighborhood. For *M*1, *AT*_*M*1_, the value is zero, because *M*1 has high privileges, and it does not wait for other’s permission. It just sends a control packet in its surroundings, as illustrated in Equation (11) as:

(10) $$AT_{M1}=0$$

(11) $$AT_{M2}=PD_{1000}+PD_{M1M2}$$

(12) $$AT_{M3}=AT_{M2}+PDC_{M2M3}$$

**Figure 8:**
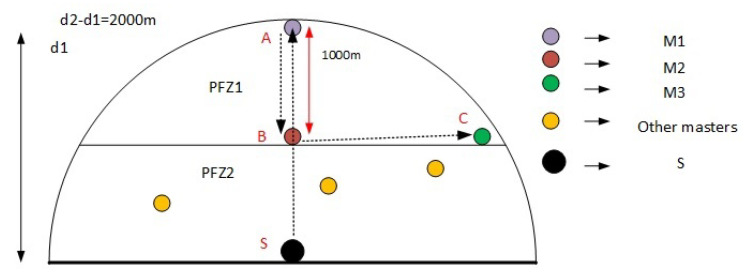
Holding time and activation time calculation.

The above equations demonstrate activation time for *M*2, where the first term is propagation time taken by a data packet to travel over 1000 m, and the other term is for propagation delay of the data packet from *M*2 to *M*3. If we closely look at Figure 8, it demonstrates the first term i.e., when node S sends data packet in its neighborhood, node B receives data packet far earlier than node A. Similarly, the *AT*_*M*2_ and for activating *M*2, it must wait for the data packet to reach M1 and for the control packet to travel from *M1* to *M2*. Equation (13) shows the activation time for *M2*, whose 1st term is the activation time of *M2*, while the 2nd term is the control packet’s propagation delay from *M2* to *M3*. Activation time contributes a little to the E2E delay of the whole network.

#### 4.11.2. Holding Time (HT) Calculation

Holding time is the time slot for which a node possesses a data packet and the time in which it broadcasts the control packet in the range of 2000 m. It is known that all nodes have the data packet, but they are bounded to hear for control packet call from their priority master node. This call is only in the case when the former node is ready to send the packet forward. If any of these masters do not receive the control packet from their ex-master, it means the master that is next in the priority list is now eligible for forwarding the packet. This transfer of control on the master’s level only happens whenever a node encounters an energy hole or void hole on its way. Equations (14)–(16) are the holding time equations for *M1*, *M2*, and *M3*, respectively.

(13) $$HT_{M1}=AT_{M1}+PDC_{2000}$$

(14) $$HT_{M2}=AT_{M2}+PDC_{2000}$$

(15) $$HT_{M3}=AT_{M3}+PDC_{2000}$$

### 4.12. Data Delivery Scheme

**Figure 9:**
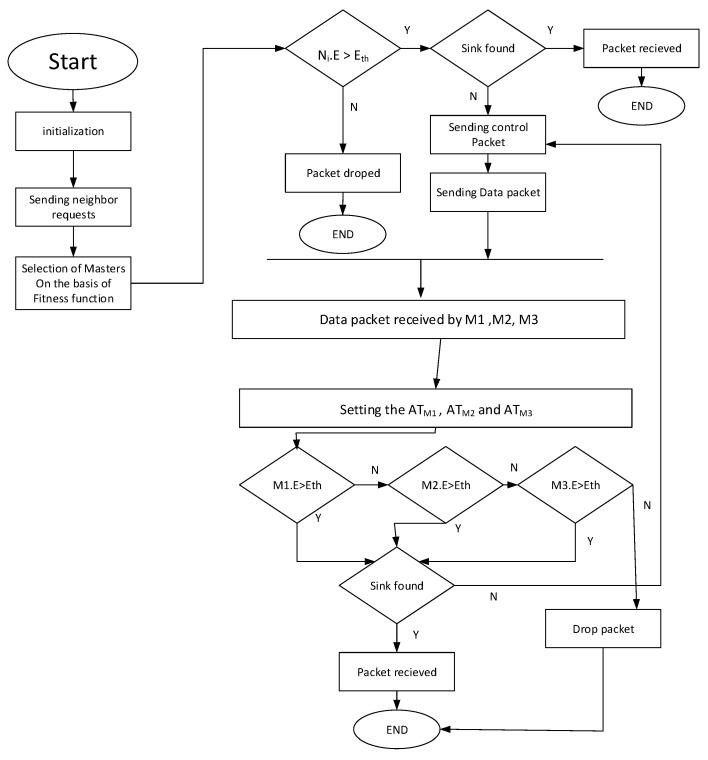
Flow Chart of the whole process.

For the process of packet generation and packet forwarding, we follow a certain data delivery scheme. In this scheme, every node can generate packets (NR, ACK, CTRL, DATA) in the whole process. When a node becomes a source (i.e., node that holds/generates the DATA packet), it will first check the sink in its range for its existence, and then handover the DATA packet to sink without following the forwarding scheme. If there is no sink in its proximity, it follows the scheme in which the nodes are in the neighborhood. The nodes are divided into Suppressed and Potential Forwarding Nodes (PFN) based on depth greater than and less than the source node, respectively. The PFNs are further subdivided into masters and slaves based on MSF values. The MSF values are calculated on each PFN using Equation (10). The source node fetches these values from the PFNs through the neighbor request packet and evaluates the PFNs into masters and slaves based on higher and lower values, respectively. Algorithm 1 plays a major role in selecting the desired best master nodes for the forwarding of the data packet. Slave nodes are suppressed by the CTRL packet broadcast by the source node, containing the IDs of the masters. After the mentioned selection process, the clocks are allocated to the master nodes using the activation time and holding time Equations (10)–(15). The node that beats the timer forwards the data and then follows an Algorithm 2 and the flowchart in Figure 9, for forwarding data until it finds the sink, and marks the successful data delivery to the sink.

### 4.13. Restricting Extra Reception of Packets

**Figure 10:**
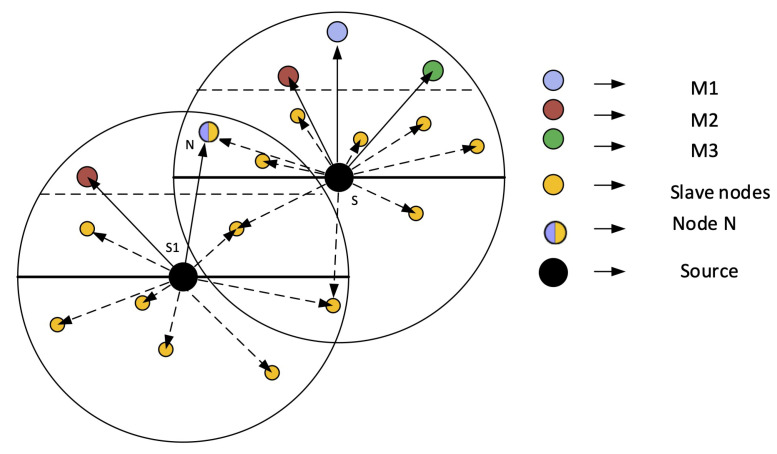
Restricting extra reception of data packets.

Our approach minimizes the data packet reception in the PFNs of a node that is not eligible to send data (i.e., slave nodes). In prior routing protocols, all PFNs receive the data packet; however, eventually, further transmission is carried out by one of them. Since we are focusing on optimizing our network in terms of energy, we cannot afford wastage of energy in the form of receiving energy. Therefore, we adopted a method that suppresses all nodes, ineligible for forwarding the data packet to save the receiving energy. One possible scenario is depicted in Figure 10. As shown in the figure, a node suppressed by the sender node may also reside in some other node’s range, while this node is the master node of its own PFN i.e., Node N is the slave node of Node S1 but the master node of S and therefore can receive data packets from S1 but not from S.
**Algorithm 2:** Packet’s transmission Algorithm
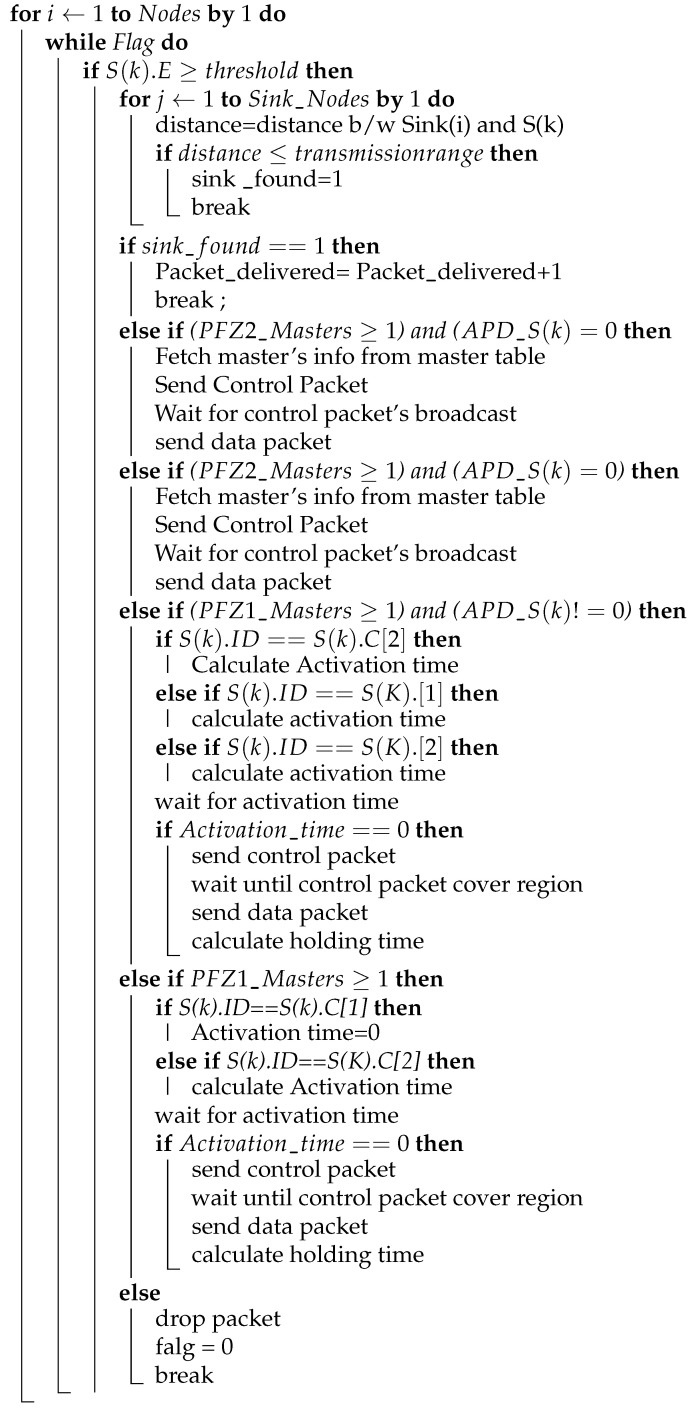


## 5. Simulation

### 5.1. Simulation Setup

**Figure 11:**
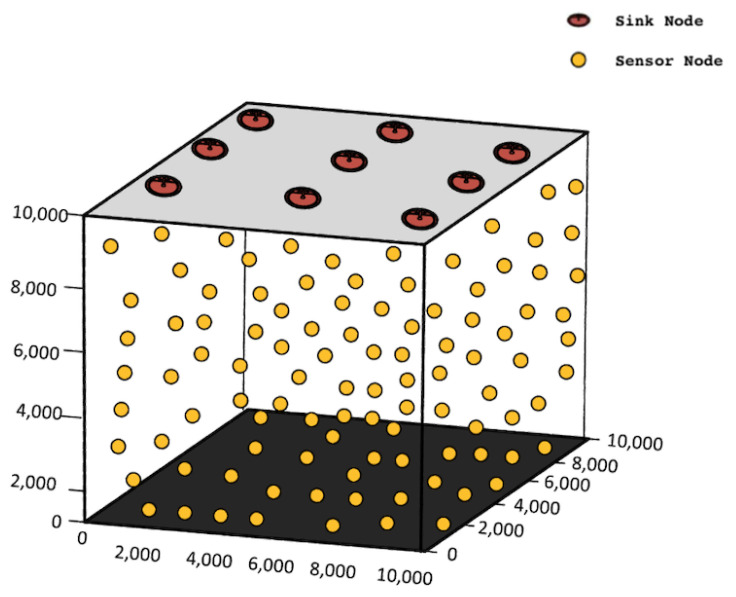
Network deployment.

In this section, we thoroughly describe the simulations that we carried out using MATLAB software. Simulations are based on the comparative analysis of WDFAD with our proposed protocol (ERPMSA-UWSN) based on varying parameters i.e., PDR, energy tax, end-to-end delay, and the different number of sinks. Furthermore, most of the environmental parameters and settings are taken from the WDFAD protocol system setup. The 100–500 sensor nodes are randomly deployed in the 3D orientation inside water and over 10 Km^3^ volumetric space, as shown in Figure 11. The individual average coverage of a node ranges from 2 km^3^ to 10 Km^3^ in a sparse to denser network, respectively. Multiple sinks are deployed on the ocean surface stationary and set to receive data from relay nodes. These randomly deployed nodes follow the random walk mobility model (RWM) [44] for movement. They select their next direction based on random probability and move there with the random speed ranges from 1 m/s to 5m/s along the X-Y plane. They change their position after every 2 s and the RWM model keeps the node bounded in coverage boundary. The movement of the nodes is mostly considered in the horizontal axis, while along the vertical axis it is considered slightly. Data packet size taken is 83 Bytes (72 Bytes payload and 11 Bytes header), while other packets (i.e., neighbor request, acknowledgment, control packet) size is 50 bit (6 Bytes) each, with the data rate of 16Kbps. The transmission range of every node is at least 2 Km^3^, the initial energy of every node is 100 J and the threshold energy (i.e., the minimum amount of energy that a node needs to stay alive) is 5 wJ. Furthermore, the acoustic signal speed is 1500 m/s, while the bandwidth of the medium is 4000 Hz. Since we perform simulations in deep and shallow water, hence we must categorize the ocean based on depth. We figured out from the review study that up to 100 m depth the ocean is shallow, while onward 100 m it is deep. Therefore, we deploy the source node in the region of 10 km^3^ so that it covers both the shallow and deep domains of the ocean. The parameters under which we performed the simulation are listed in Table 3.

### 5.2. Performance Breakdown and Comparison

We evaluate ERPMSA-UWSN performance in comparison with WDFAD-DBR based on performance metrics, i.e., the Packet Delivery Ratio (PDR), an End-to-End Delay (E2ED), energy tax, and the Accumulated Propagation Distance (APD).

#### 5.2.1. Packet Delivery Ratio (PDR)

The packet delivery ratio is associated with the successful delivery of packets to the sink. PDR indicates the ratio of the packets count which reach their destination (Sink) and the total packets generated by the network. The duplicate packet is considered to be a single packet, while redundancy is not allowed.

(16) $$PDR=\frac{Packets\;received}{Packet\;Sent}$$

#### 5.2.2. Energy Tax

It is the total average amount of energy required per node to send a DATA packet to its destination. It includes the sending and receiving energy as well as the processing, computational and idle state energies.

(17) $$Energy-Tax=\frac{E_{total}}{nodes*packets}$$

where *E_total_* is the energy expenditure, *nodes* are the total number of nodes in the network and *packets* are that which have successfully found their destination.

#### 5.2.3. End-to-End Delay (E2ED)

End-to-end delay measures the average time from the instant of the packet generation to the moment it reaches to sink. Due to the multiple sink architecture, there is a possibility to receive the same packet by more than one sink. In this case, the E2ED of that node is considered, whose packet finds the sink earlier.

#### 5.2.4. Accumulated Propagation Distance (APD)

The mean Accumulated Propagation Distance (APD) is equivalent to the mean accumulated distance covered by the successfully delivered packets on each hop. In this multi-sink architecture, there is a case where more than one sink receives the same packet. Hence in such a scenario, the shortest Accumulated Propagation Distance is considered to be the APD of that specific packet, while the APD is computed through the given equation as shown as:

(18) $$APD=\frac{1}{n-p}\sum_{j=1}^{n-p}\sum_{i=1}^{h}dist_{i}^{j}$$

where the number of packets are denoted by *n*−*p*, *h* pertains to the hop count of the packet from the source node to the sink and $Dist_{j}^{i}$ is the distance measured from the *i*th hop to the *j*th packet.

### 5.3. Simulation Results

Simulations are performed in MATLAB (2017a) on the theoretical data to get the plots of the performance metrics to evaluate whether our proposed work is contributing to the main cause of saving energy or not. The main focus is to prolong the network life by saving energy as much as possible; however, we dealt with some trade-offs and compromised some metrics (E2ED and APD) to gain improvements in PDR and energy tax. We compare ERPMSA-UWSN and WDFAD-DBR based on different parameters including sink numbers variation and node density.

#### 5.3.1. Simulation of ERPMSA-UWSN in Comparison with WDFAD

**Figure 12:**
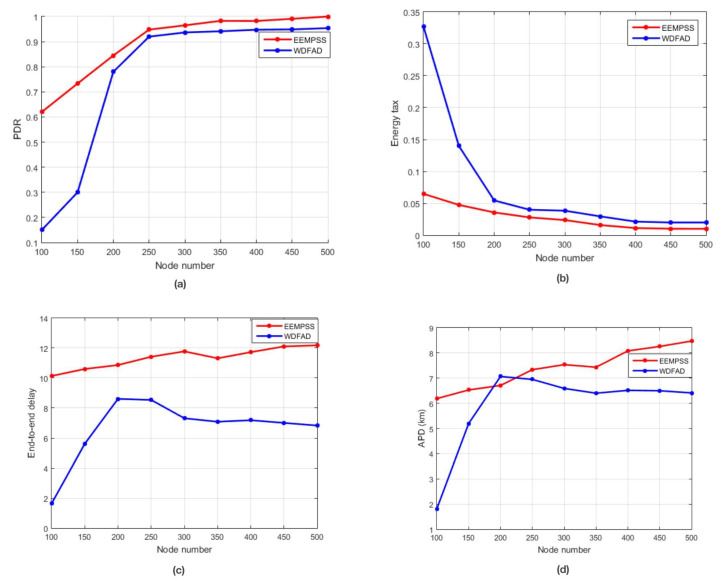
(**a**) PDR against the number of nodes; (**b**) energy tax versus the node count; (**c**) end-to-end delay against number of nodes; (**d**) APD compared to the node count. Comparison of ERPMSA-UWSN with WDFAD.

**Figure 13:**
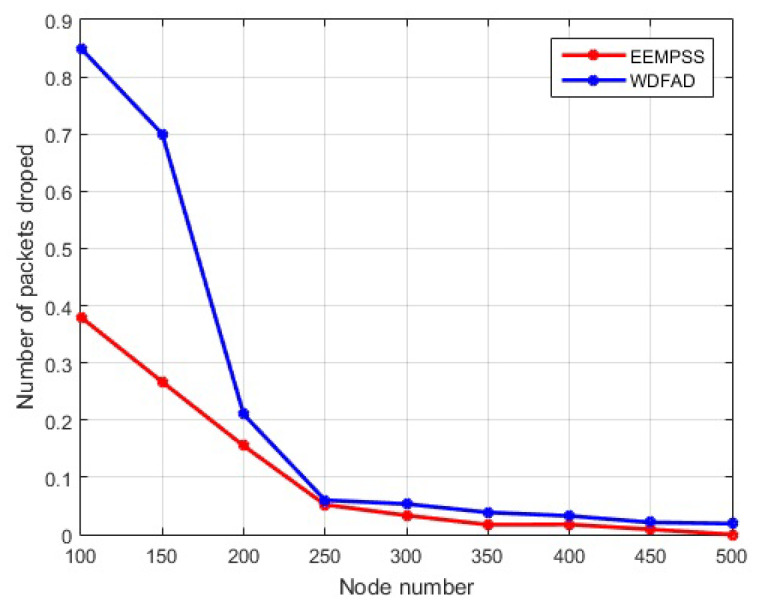
Packet drop profile.

As shown in Figure 12a, the PDR increases with an increase in the number of nodes for both WDFAD-DBR and ERPMSA-UWSN. This is because the increase in the network density also increases the probability of active node occurrence, and reducing the void hole occurrence. ERPMSA-UWSN outclassed WDFAD in terms of PDR in both sparse as well as dense networks. WDFAD selects the next forwarding node based on the difference in weighting depth between the two hops. Alternatively, ERPMSA-UWSN selects the next forwarding node i.e., the master node based on the depth difference with higher energy and a greater number of PFNs. There are two types of holes in the network—, one is called void hole, while the other is referred to as energy hole. The void hole occurs due to the absence of PFNs in the forwarding area, while the energy hole exists due to the lack of sufficient transmission energy in the potential forwarding node, as shown in Figure 12. The difference of PDR between ERPMSA-UWSN and WDFAD decreases from sparse to dense networks due to the network density. Additionally, WDFAD drops the packet in the absence of PFN in its range i.e., void hole, but ERPMSA-UWSN keeps track of the void hole at the time of selecting the master node for transmission. Therefore, it rarely encounters void holes in its transmission period; however, sometimes there is a chance of an energy hole that occurs due to the additional use of the node. This is also resolved by adopting the 2nd or 3rd master in the region. In case, when the 2nd and 3rd master do not exist in the vicinity, we divide the PFZ into two portions, the lower PFZ2 portion which is denser in node population, and the upper PFZ1 which is mostly sparse. During the selection process, MSF filters out those masters whose PFZ1 has a minimum of 1 node, and if does not exist, a forwarding node from PFZ2 is selected. In a sparse network, WDFAD drops more packets which broaden the proportional gap between the plot of WDFAD and ERPMSA-UWSN, increases the probability of active nodes, and decreases the void holes, consequently decreases the packet drop as well as the gap in PDR results. To encounter the energy hole, our protocol provides additional support by suppressing redundant transmissions of the packet. This results in reducing the average energy consumption, which prolongs the node and network life, as a result of eliminating the energy holes.The void/energy holes and packet collision affect the network throughput and PDR. Like the WDFAD, we also use the two-hop mechanisms in our proposed protocol (ERPMSA-UWSN) to deal with the void holes. However, in ERPMSA-UWSN, it is ensured that the selected master has no void hole in its range. In addition, the problem of energy hole is significantly highlighted in ERPMSA-UWSN compared to WDFAD, as it mainly focuses on transmission energy economization. This is achieved by adapting distance-based transmission methods for packet transmission and selecting the best master for forwarding, whose responsibility is to keep the network free of energy holes. The collision of packets occurs whenever packet traffic is high since the ERPMSA-UWSN suppresses the redundant transmissions which leads the network to have low packet traffic and thus minimizes the collision of packets. For these reasons, the network throughput for our proposed protocol, in sparse as well as dense networks, is higher than the existing ones i.e., WDFAD-DBR whether sparse or dense networks. The slow growth noted in the dense network is justified because ERPMSA-UWSN has limited sources of data packet after suppressing the majority of the PFNs. In contrast to WDFAD, there are multiple packet resources in the network, which may cause a collision and cause packet loss in the network. In the case of a dense network, we produce more chance of finding the best master for the transmission, leading the network out of energy holes and void holes situations and preventing packet drop. This behavior of the PDR is well justified by the packet drop profile as shown in Figure 13. It can be seen that the amount of packet drop decreases with the increase in the number of nodes, and ERPMSA-UWSN shows less packet drop compared to WDFAD in both sparse as well as dense networks.

Next, we breakdown the energy tax of the proposed scheme (ERPMSA-UWSN) and the competing protocol (WDFAD). Transmission energy has a higher proportion in energy consumption than the other energies i.e., idle state, receiving, processing, and sensing energies. In Underwater acoustic networks, packet transmission is the costliest process, especially since the transmission of the data packet is the most energy-consuming due to its large size compared to other packets. This evaluation of energy is already validated through experimental findings in [45]. The energy cost of transmitting one single bit is approximately equal to thousands of processing [46]. The algorithm’s computational complexity is also an energy-consuming factor i.e., the cost that a network must bear for the complex computation. A decreasing trend has been noted in the energy usage with the increase in node numbers. It is because of the increased probability of a master’s abounded existence, which leads to the successful transmission of packets. Furthermore, an increase in node number also suppresses the energy wastage, caused by the re-transmission of the packet. An increase in nodes also leads to the enriched energy resources that might contribute to this decreasing trend.

The method adopted for selecting the next forwarder (master) is superficially more contributive in the campaign of energy optimization. It targets the extra transmissions of the data packet by introducing a new packet (CTRL PACK), which is smaller in size and suppresses the additional data packet transmissions. Data packet has a size of 72 bytes, while CTRL PACK has a size of 5 bytes, which makes a mighty difference in the energy equation. Master nodes have only the privilege to receive the data packet, while slaves remain unprivileged in the sense of data packet reception. In contrast, WDFAD used to send the data packet to each node that lies in its range, and at last one of them must transmit, while the remaining one must drop that packet. Saving transmission energy put a great influence on the total energy cost which results in low energy consumption.

Our scheme adopted the adaptive transmission power leveling mechanism which consequently leads to the economization of energy. As transmission energy is applied based on the displacement of the node in the transmission range i.e., farther node means more transmission power is exerted and vice versa. In contrast to WDFAD, fix the amount of transmission energy is used for packets transmission on each hop irrespective of the next forwarder displacement. In the sparse environment, this approach does not play a major role in saving energy due to the wide dispersion of the nodes in the specified range. However, in a dense network, there is a mighty part played by this approach because a greater number of nodes is present in the maximum range. In general, trend (most rare), the source node must transmit with the maximum power so that it targets maximum numbers of the nodes in its proximity. Though, in WDFAD, the source node will transmit with the maximum fixed power even its next forwarder is closer to it. The process of adaptive transmission power leveling significantly reduces energy consumption without affecting any other performance metric. Therefore, either the sparse or the dense, the novel protocol (ERPMSA-UWSN) persuasively outperformed the competing protocol (WDFAD) in terms of energy usage.

End-to-end delay is associated with the average time taken by a packet to reach its destination. This average time includes transmission time, processing time, idle state time, and propagation time. Propagation time and processing time matter in delay-tolerant protocols, for instance, the presented protocol in which propagation time and the processing time are extended due to the distance-based calculation of the holding time and activation time. In contrast, the holding time calculation of WDFAD is based on the weighting depth of the nodes, which results in a minimal end-to-end delay. The E2ED is directly associated with the behavior of the APD curve. The APD of the presented protocol is higher than the competing protocol because of the longer route followed by the packet, in search of the best forwarder in the network. Our protocol keep account of whether the packet reached the destination successfully, irrespective of the length of the route. On the other hand, WDFAD keeps the track of the route followed by the packet, which leads to low APD.

We recorded the percentage improvement in the PDR and energy tax, as listed in Table 4.

#### 5.3.2. Simulation Results Based on Sink Variation

**Figure 14:**
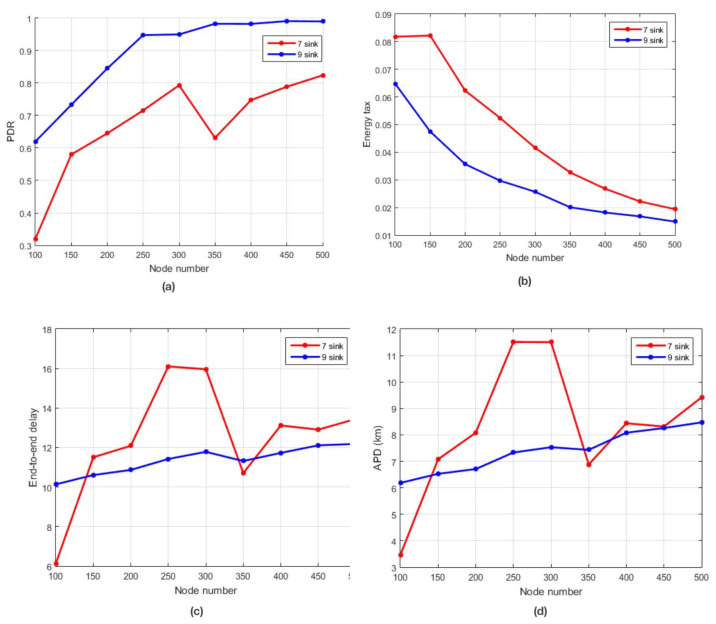
(**a**) PDR against the number of nodes; (**b**) energy tax in comparison to the number of nodes; (**c**) end-to-end delay against the number of nodes; **(d)** APD vs. number of nodes. Comparison of ERPMSA-UWSN for 7 and 9 sinks.

To investigate the best economical options, we analyzed our protocol’s performance with the standard number of sinks (7) as well as a higher number (9) used by the existing WDFAD-DBR. Our proposed protocol does not perform well for 7 sinks as compared to 9 sinks, in both sparse and dense networks. This behavior is obvious as by decreasing the number of receivers in a network reduces the network’s performance. Consequently, the load on a single receiver increases which results in the increase of packets collision. Furthermore, this results in a lower PDR, higher energy tax, E2ED, and a higher APD, as shown in Figure 14a–d, respectively. Another limitation is the slow growth noted in a dense network. It is because ERPMSA-UWSN has limited sources of data packets after suppressing the majority of the PFNs, in contrast to WDFAD. The possibility of a void/energy hole and/or the absence of the potential forwarding node in the transmission range still exists.

## 6. Conclusions and Future Work

In this paper, we proposed a novel protocol, called Efficient Routing Protocol based on Master–Slave Architecture for Underwater Wireless Sensor Network (ERPMSA-UWSN) that aims to optimize energy consumption in the network. The routing scheme of ERPMSA-UWSN is based on several parameters including the neighboring nodes, hops count, energy level, master selection function, and holding time. We compared the performance of our proposed protocol with the closely related state-of-the-art Weighting Depth Forwarding Area Division Depth-Based Routing (WDFAD-DBR) protocol. Our simulation results demonstrated that ERPMSA-UWSN performs better than the WDFAD-DBR, i.e., an improvement of 13% on energy tax and 4.8% on PDR, on average, is achieved. In addition, the hidden terminal problem and the traffic overhead are controlled by suppressing the reproduction of data packets in the network which reduces the energy consumption. The existence of an energy hole or the absence of the potential forwarding node in the transmission range is still a challenging parameter resulting in slightly decreased reliability.

In future, sinking mobility can be added to the proposed routing scheme that may result in further decreasing the Accumulated Propagation Distance (APD) and End-to-End delay.

## Figures and Tables

**Table 1:** Comparison of existing UWSNs protocols.

Protocol Category	Protocol	Advantages	Drawbacks
Localization-Based Routing	VBF [4]	Limiting the direction and forwarding range	Cause duplicate packets in dense and void holes in sparse network
	HH-VBF [5]	Improved PDR	Fails to provide energy fairness and void holes avoidance
	AHH-VBF [6]	Energy efficiency is achieved due to power adjustment	Imbalanced energy consumption
	AVN-AHHVBF [7]	Energy efficiency	High packet loss
	ESEVBF [8]	PDR and energy optimization	Fails in sparse network
	NEFP [9]	Energy efficiency	does not perform well in sparse network
	TC-VBF [10]	Energy efficiency	Network throughput is lower for fewer nodes
	MEES [11]	Energy optimization, energy balancing	packets are dropped by nodes for out-of-range sinks
	DTMR [12]	Network throughput is higher, lower delay	Unreliable for direct transmission
	FVBF [13]	Energy optimization, delay tolerant and higher throughput	Lower network lifetime due to rapid dying of nodes in the pipe
Localization-Free Routing	DBR [14]	Increase in PDR in dense network	Bad performance in sparse network
	DSRP [15]	High throughput	High energy consumption and end-to-end delay
	ODBR [16]	prolong network life, energy balancing	Not good for in-depth water regions as the nodes located at the bottom needs higher energy for sending attributes
	EBECRP [17]	Energy efficiency and network balancing	Higher packet drop ratio because of the cluster heads mobility or death
	Hydrocast [18]	Energy efficiency	Does not perform well in sparse network, increased load because of using opportunistic routing
	WDFAD-DBR [19]	Increase in network lifetime and decreased	energy consumption E2ED is increased
	DOW-PR [20]	Increase in PDR and Decreased in Energy in energy consumption	Increase in E2ED
	OMR [24]	Higher network throughput, lower E2ED, energy optimization	Does not perform well for long-range communication because of the fading issue of RF waves
	QERP [25]	Increase in PDR and Decreased in Energy in energy consumption and low packet latency	Earlier death of cluster heads because of overloading leading to the creation of energy holes
	EECOR [26]	Energy efficiency, high throughput	Increased delay due to the communication among various nodes in forwarding region
	RRSS [27]	Energy efficiency	Overloaded nodes because of more data in the vector
	UMDR [28]	Energy optimization, higher throughput, and lower E2ED	complications due to the frequent calculations of the next hop and information about antenna

**Table 2:** Symbols/Abbreviations.

Symbols/Abbreviations	Description
UWSN	Underwater Sensor Network
TWSN	Terrestrial Wireless sensor network
MSF	Master Selector Function
NR	Packet for Neighbor request
DATA	Data packet
ACK	Acknowledgment
CTRL	Control Packet
NTAB	Neighbor Table
MTAB	Table for the master nodes
PFZ	Potential Forwarding Zone
PFZ1	cap of the upper hemisphere
PFZ2	lower Part of upper hemisphere
PFN	Potential Forwarding Node
AT_Mi_	activation time for Mi i = 1,2,3.
HT_Mi_	Holding time for Mi i = 1,2,3.
PDC	Propagation delay for control packet
APD	Accumulated Propagation Distance
PDR	Packet Delivery Ratio
En	Eligible Neighbor
E_th_	Threshold energy for data transmission
N_i_.E	Energy of node i (i = 1,2,3,4...)

**Table 3:** Simulation parameters.

Parameters	Values
Node count	100:50:500
Aggregate of sinks	9
Maximum range of transmission per node	2 Km
Deployment region: 3D area of 10 Km	10 Km^2^
DATA Header size	11 Bytes
DATA Payload size	72 Bytes
Size of ACK Packet	50 bits
Size of neighbor request	50 bit
Data rate	16 Kbps
Initial energy per node	100 J
Maximum Transmission Power	90 dB re
Power threshold for receiving	10 dB re
Sending energy	50 W
Receiving energy	158 mW
Idle Energy	158 mW
Center Frequency	12 KHz
Acoustic Propagation	1500 m/s
δ	2 Km
Bandwidth	4 KHz
Random Walk	2 m/s
Probability of moving left	0.5
Probability of moving right	0.5
Alive node threshold energy	5 J
P_1_	1
P_2_	0.5

**Table 4:** Qualitative analysis of the ERPMSA-UWSN based on PDR and energy tax with WDFAD.

Number of Nodes	% Improvement in PDR	% Improvement in Energy Tax
100	26	47
150	9	43
200	2	6
250	1	2
300	1.5	3
350	1.4	4.1
400	1.1	3.5
450	0.98	4.2
500	0.9	4.6
Total Avg	4.8	13
